# Difluorinated-Curcumin **(**CDF**)** Restores PTEN Expression in Colon Cancer Cells by Down-Regulating miR-21

**DOI:** 10.1371/journal.pone.0068543

**Published:** 2013-07-24

**Authors:** Sanchita Roy, Yingjie Yu, Subhash B. Padhye, Fazlul H. Sarkar, Adhip P.N. Majumdar

**Affiliations:** 1 Department of Veterans Affairs Medical Center, Wayne State University, Detroit, Michigan, United States of America; 2 Department of Internal Medicine, Wayne State University, Detroit, Michigan, United States of America; 3 Department of Pathology, Karmanos Cancer Institute, Wayne State University, Detroit, Michigan, United States of America; 4 Department of Oncology, Karmanos Cancer Institute, Wayne State University, Detroit, Michigan, United States of America; 5 ISTRA, Department of Chemistry, MCE Society′s Abeda Inamdar Senior College of Arts, Science and Commerce, Pune, India; Baylor University Medical Center, United States of America

## Abstract

Despite recent advancement in medicine, nearly 50% of patients with colorectal cancer show recurrence of the disease. Although the reasons for the high relapse are not fully understood, the presence of chemo- and radiotherapy-resistant cancer stem/stem-like cells, where many oncomirs like microRNA-21 (miR-21) are upregulated, could be one of the underlying causes. miR-21 regulates the processes of invasion and metastasis by downregulating multiple tumor/metastatic suppressor genes including PTEN (phosphatase and tensin homolog). Tumor suppressor protein PTEN controls self-renewal of stem cells. Indeed, our current data demonstrate a marked downregulation of PTEN in SCID mice xenografts of miR-21 over-expressing colon cancer HCT116 cells. Colonospheres that are highly enriched in cancer stem/stem like cells reveal increased miR-21 expression and decreased PTEN. Difluorinated curcumin (CDF), a novel analog of the dietary ingredient curcumin, which has been shown to inhibit the growth of 5-Flurouracil + Oxaliplatin resistant colon cancer cells, downregulated miR-21 in chemo-resistant colon cancer HCT116 and HT-29 cells and restored PTEN levels with subsequent reduction in Akt phosphorylation. Similar results were also observed in metastatic colon cancer SW620 cells. Since PTEN-Akt confers drug resistance to different malignancies including colorectal cancer, our observation of normalization of miR-21-PTEN-Akt pathway by CDF suggests that the compound could be a potential therapeutic agent for chemotherapy-resistant colorectal cancer.

## Introduction

The emerging challenge in the treatment of colorectal cancer (CRC), the third most common cancer, is the relapse of the disease. Nearly 50% of CRC patients show recurrence of the disease. The presence of chemo- and radio-therapy resistant cancer stem/stem-like cells (CSCs/CSLCs) could be one of the underlying causes [1]. These cells are small population of undifferentiated tumor initiating cells having infinite self renewal capacity and are therefore referred to as tumor initiating cells [2]. PTEN (phosphatase and tensin homolog), a tumor suppressor gene has been reported to play role in stem cell self-renewal [3]. PTEN acts as a lipid phosphatase to dephosphorylate phosphatidylinositol 3,4,5-trisphosphate (PIP3), antagonizing the PI3-K/Akt pathway. Downregulation of PTEN in different human tumors has been shown to result in resistance to conventional therapy and recurrence of cancer after initial treatment [4]. The PTEN-Akt pathway confers drug resistance to different malignancies including colorectal cancer [5], [6], [7], [8].

PTEN is one of the target proteins of miR-21, which is upregulated in many malignancies, including colorectal cancer [9]. microRNAs negatively regulate the expression of target genes by cleaving mRNA or through translation repression [10]. miR-21 not only regulates tumor growth but also invasion and metastasis by targeting multiple tumor/metastatic suppressor genes such as PTEN [11]. We have reported that 5-Fluorouracil and Oxaliplatin (FU-Ox) resistant [chemo-resistant (CR)] colon cancer HCT116 and HT29 cells exhibit enrichment of CSCs/CSLCs and elevated levels of mature miR-21 and that miR-21 induce stemness in colon cancer cells [12]. These observations prompted us to search for agent(s) that would modulate cellular events that are associated with miR-21-induction of stemness in colon cancer cells.

Difluorinated curcumin (CDF), a nontoxic analog of the dietary ingredient curcumin with a much greater bioavailability than the parent compound [13], [14], is one such compound. CDF has been shown to modulate the expression of miR-21 and PTEN in pancreatic cancer [15], [16], [17], [18] and to inhibit the growth of CR colon cancer cells, and induces disintegration of colonospheres that are highly enriched in CSCs/CSLCs [19]. Additionally, we found CDF to up regulate the expression of miR-34 [20], which is reported to be downregulated in colon cancer [21]. To determine whether the CDF mediated inhibition of growth of CR colon cancer could partly be attributed to restoration of miR-21-PTEN-Akt axis, we have examined (a) the relationship between miR-21 and PTEN and (b) the effect of CDF on the expression of miR-21, PTEN and activation of Akt axis in different colon cancer cells.

## Materials and Methods

### Cell lines and culture condition

The human colon cancer cells HCT-116 (p53 wild type; *K-ras* mutant), HCT-116 (p53 null; *K-ras* mutant), HT-29 (p53 mutant; *K-ras* wt) and SW620 were obtained from the American Type Culture Collection (ATCC, Rockville, MD). Chemo-resistant (CR) colon cancer cells were generated in our laboratory as described previously [12]. Briefly, HCT116 and HT29 cells were incubated with a clinically relevant dose of FU-Ox (25 μM of 5-FU and 0.625 μM of oxaliplatin) for 72 hours. The medium was removed and the adherent cells, that survived the FU-Ox insult, were cultured in DMEM containing 10% FBS without the drugs for 3 to 4 days. The add-remove FU-Ox cycle was repeated 12 times. The surviving cells were then passaged and exposed to higher doses of combination of FU-Ox (100 µM of 5-FU + 2.5 µM of oxaliplatin) for 2–3 weeks. Finally, the chemo-resistant cells were maintained in normal culture medium containing 50 μM 5-FU + 1.25 μM oxaliplatin. SW620 colon cancer cell line was maintained in RPMI, supplemented with 10% fetal bovine serum (FBS).

Colonospheres were generated from parental HCT116 and HT29 cell lines using DMEM/F12 (1:1) media supplemented with B27 (Life Technologies, Gaithersburg, MD), as reported previously [12] [22]. Briefly, the cells were allowed to grow in non-differentiating condition (stem-cell media) in ultra low-attachment plates (Corning Inc, Lowell, MA) for 10–14 days. The stem cell medium was supplemented with B27,, 20 ng/ml EGF (Sigma, St Louis, MO), 10 ng/ml fibroblast growth factor (Sigma), and antibiotic/antimycotic. By the end of 5 days, only the stem-like cells were able to survive in non-adherent conditions while the non-stem like cells died off. Fresh stem cell media was provided to the surviving cells every 5 days. The spheroids formed were photographed at 10× magnification. The cells were passaged using 0.05% trypsin-EDTA (Invitrogen) when confluent.

### Treatment of colon cancer cell lines

CDF was dissolved in DMSO. Where applicable, CR HCT116, CR HT29 or SW620 cells were treated with 100 nM CDF for 72 hours. Control cells received 0.05% DMSO. After 72 h, the cells were subjected to DNA, RNA and protein isolation.

### Xenograft tissue specimens

The animal research protocol was approved by Wayne State University Institutional Animal Care and Use Committee (IACUC). Xenograft derived from HCT116 cells, stably transfected either with miR-21 or the vector (pcmv), were generated in SCID mice as reported earlier [12]. Formalin-fixed paraffin-embedded (FFPE) xenograft tissues were used to isolated RNA using miRNeasy FFPE Kit (Qiagen) as described previously [20].

### Real-time RT-PCR

RNA was isolated from FFPE tissues using miRNeasy FFPE Kit (Qiagen) and from cultured cells using miREasy kit (Qiagen). Twenty nanogram of total RNA were reverse transcribed into cDNA using Universal cDNA Synthesis Kit (Exiqon, Woburn, MA) according to the manufacturer's protocol. To estimate the relative levels of PTEN-mRNA, 500 ng of RNA were reverse transcribed into cDNA using Gene Amp Gold RNA PCR Kit (Applied Biosystems). Real time PCR was performed using specific primers for miR-21 (Exiqon) and PTEN [23] along with SYBR^®^ Green PCR Reagents (Applied Biosystems). The relative amount of miR-21 and PTEN-mRNA were normalized to RNU1α (Exiqon) and human β-actin gene respectively.

### Western blot analysis

Western blot analysis was performed according to our standard protocol [22]. In brief, the cells were lysed; the protein concentration was determined by the Bio-Rad Protein Assay kit (Bio-Rad). Lysates were electrophoresed by SDS-PAGE and the proteins transferred onto polyvinylidene difluoride (PVDF) membranes (Millipore) followed by blocking with BSA at room temperature for 1 h. They were then incubated with primary antibodies overnight at 4°C, subsequently washed and incubated with compatible secondary antibodies. The protein bands were visualized by ECL prime western blotting detection reagent (GE Healthcare Biosciences). The membranes were stripped as needed for further probing.

### Luciferase Assay

HCT116-CR cells were transfected either with anti-sense miR-21 or negative control [NC] ( Ambion) along with the luciferase reporter plasmid having PTEN 3'UTR seed sequence for miR-21 [11] and pMIR-REPORT b-gal control plasmid DNA (Applied Biosystems) using Lipofectamine^TM^ 2000 according to the manufaturer's protocol. Six hours post transfection, the media was changed and the cells transfected with NC were incubated with 100 nM CDF for 24 h. Luciferase activity was determined using Promega luciferase kit following manufaturer's protocol. β-galactosidase assays were performed to normalize the transfection variability. The data were presented as relative luciferase activity per microgram protein.

### Statistical analysis

The results were expressed as means ± SD. Comparisons of the continuous variables between two independent groups were calculated using two-tailed student's t test. The p value of <0.05 was considered to be statistically significant.

## Results

### PTEN expression was downregulated in miR-21 over- expressing xenograft

**Figure 1:**
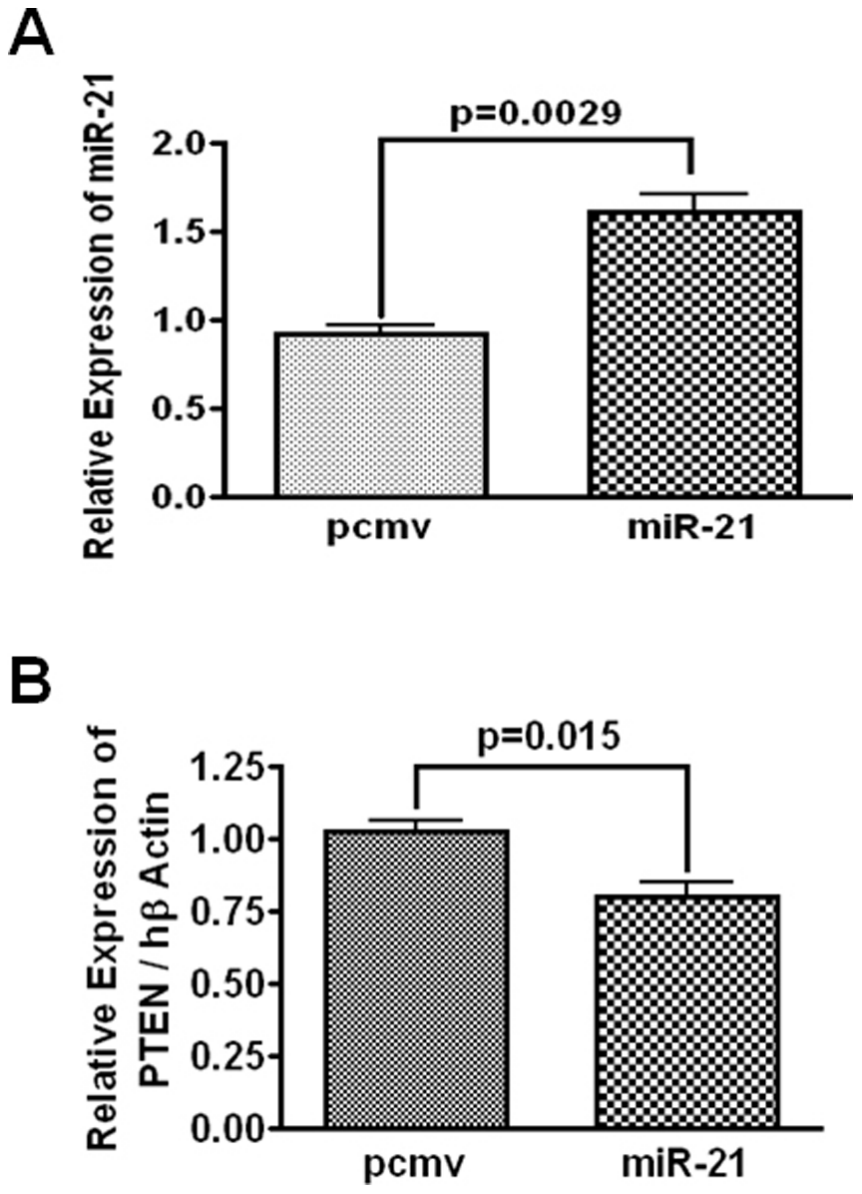
miR-21 (A) was upregulated and PTEN (B) was downregulated in xenografts, derived from miR-21 over-expressing HCT116 cells (miR-21) and corresponding vector controls (pcmv) in SCID mice as described previously [12]. Quantitative real-time RT PCR was performed with RNA isolated from formalin fixed paraffin embedded xenografts.

PTEN is a target of oncomir miR-21. To examine the relationship between miR-21 and PTEN, SCID mice xenografts derived from miR-21 over-expressing HCT116 cells (miR-21) and the vector-transfected control cells (pcmv) were analyzed for the expression of miR-21 and PTEN. As expected, the data from qRT-PCR analysis revealed that the relative levels of miR-21 were significantly higher in xenografts from miR-21 over-expressing HCT-116 cells, compared to the controls (Fig. 1A). In contrast, PTEN mRNA levels in xenografts from miR-21 over-expressing cells were significantly decreased compared to the controls (Fig. 1B). Taken together, the results show an inverse relationship between miR-21 and PTEN. This observation is similar to what we noted earlier for PTEN protein levels in miR-21 hyperexpressing colon cancer HCT-116 cells (12).

### PTEN-Akt pathway is activated in colonosphere

**Figure 2:**
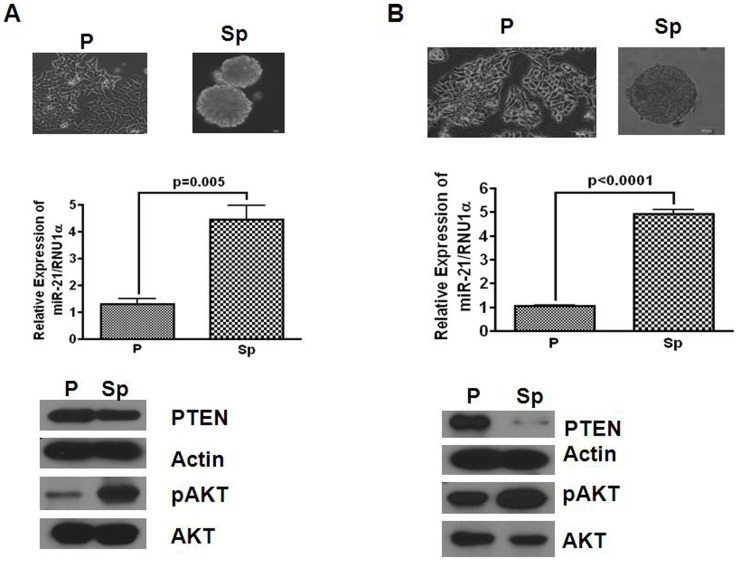
Photomicrograph of attached parental (P) and floating colonospheres (Sp) of HCT116 (A) and HT29 (B) colon cancer cells (upper panel); the middle panel represents the results of quantitative real time RT-PCR showing upregulation of miR-21 in colonospheres (Sp) when compared with the corresponding parental control (P), and western blot (lower panel) showing the relative expression of PTEN, pAKT, total Akt in parental cell and their corresponding colonospheres.

Colonospheres, considered to be surrogate tumors, are highly enriched in CSCs/CSLCs [22]. To determine whether miR-21 and its target protein PTEN are involved in regulating the functional properties of colonospheres, expressions of miR-21, PTEN and Akt, the downstream effectors of PTEN were analyzed in colonospheres generated from HCT116 and HT29. Both HCT116 and HT29 cell lines formed well-rounded spheroids (Figs. 2A & B; upper panel). Expression of miR-21, as determined by qRT-PCR, was found to be 4-5-fold higher in colonospheres, formed by both cell lines, compared to their corresponding parental cells (Figs. 2A & 2B; middle panel). On the other hand, Western-blot analysis revealed decreased expression of PTEN in colonospheres, compared to the corresponding parental cells (Figs. 2A & 2B; lower panel). As expected, reduction in PTEN in colonospheres was associated with increased activation of Akt, as demonstrated by marked increases in relative concentrations of p-Akt when compared with the corresponding parental cells (Figs. 2A & 2B lower. panel). Taken together, the results suggest that an increase in miR-21 in colonospheres that leads to reduction in PTEN activates Akt signaling pathway, which may play a role in regulating the tumorigenic properties of colonospheres that are enriched in CSCs/CSLCs.

### CDF restores PTEN expression in colon cancer cells

**Figure 3:**
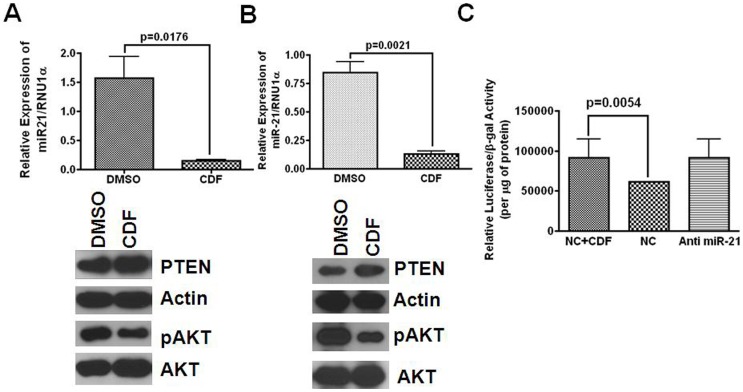
Quantitative real-time RT-PCR (upper panel) showing downregulation of miR-21 in 5-FU+Oxaliplatin (chemo-resistant) colon cancer (A) HCT116 and (B) HT29 cells in response to CDF for 72 hours. Controls were incubated with DMSO. Western-blot (**lower panel**) showed changes in the relative concentrations of PTEN, pAkt and total Akt in chemo-resistant HCT116 and HT29 cells in response to CDF or DMSO. (**C**) CDF treatment or anti-miR-21 transfection increases luciferase activity in chemo-resistant HCT116 cells transfected with pGL3-luc construct having PTEN-miR21 seed sequence. NC  =  Negative Control.

Earlier, we demonstrated that CR colon cancer cells are not only enriched in CSCs/CSLCs but also express significantly higher levels of miR-21 than their parental counterparts [12]. Since CDF has earlier been shown to inhibit the growth CR colon cancer HCT116 and HT29 cells, prompted us to determine whether CDF-induced growth inhibition of CR colon cancer cells could be attributed to restoration of miR-21-PTEN-Akt axis. Indeed, the expression of miR-21 in CR HCT116 and HT29 cells was markedly decreased when they were incubated with 100 nM CDF, compared to the corresponding DMSO controls (Figs. 3A & 3B; upper panel). This was associated with increased expression of PTEN and reduction in pAkt (activated form) in CR HCT116 and HT29 cells (Figs. 3A & 3B; lower panels); no apparent change in non-phosphorylated (total) form of Akt in either CR HCT116 or CR HT29 cells was observed in response to CDF (Figs. 3A and 3B; lower panels). The effect of CDF on miR-21 induced PTEN expression was reconfirmed by luciferase assay. CR-HCT116 cells were transfected with luciferase reporter construct containing PTEN 3'UTR miR-21 seed sequence and subsequently incubated with 100 nM CDF for 24 h. CDF significantly increased luciferase activity, over the control (Fig. 3C). For purposes of comparison, CR-HCT116 cells were transfected with anti-sense-miR-21, which revealed an increase in PTEN-luciferase activity (Fig. 3C). Collectively, the data reveal that in CR colon cancer cells, CDF down-regulates miR-21 resulting in upregulation of PTEN that leads to decreased activation of Akt. These observations suggest that CDF restores the miR-21-PTEN and Akt axis in chemo-resistant (CR) colon cancer cells.

**Figure 4:**
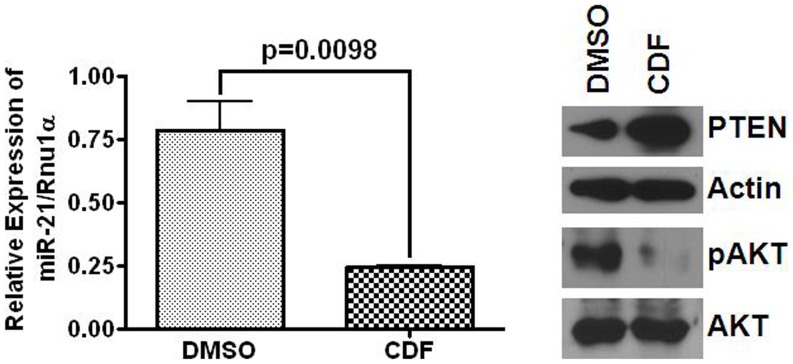
Quantitative real-time RT-PCR (left panel) showing downregulation of miR-21 in response to CDF for 72 h in SW620, a metastatic colon cancer cell line; while control was incubated with an equivalent volume of DMSO. Western-blots (**right panel**) showing changes in relative expression of PTEN, pAkT and total Akt in SW620 cells following 72 h incubation with CDF or DMSO (controls). Human β-actin served as the loading control.

To further examine whether CDF would affect the expression of miR-21- PTEN – Akt axis in metastatic colon cancer, we carried out the same experiment with SW620 cell line that was generated from metastasis colon cancer tissue. As has been observed for CR-HCT116 and HT29 cells, CDF caused a marked reduction in miR-21 expression in SW620 cells (Fig. 4). This was associated with increased PTEN levels and reduction in phosphorylated form of Akt, when compared with the corresponding controls (Fig. 4).

## Discussion

Despite recent advancement in medicine, nearly 50% of patients with CRC show tumor recurrence. Although the reasons for this are not fully understood, it may partly be related to the inability to target CSCs/CSLCs that express cancer stem cell markers, retain the limitless potential to regenerate and are resistant to conventional therapies [24] resulting in chemotherapy-refractory tumors. This would explain why it is difficult to completely eradicate cancer and why recurrence is an ever-present threat.

Although it is widely believed that cancer stem cells arise from normal stem or progenitor cells upon mutation [24], a better understanding of the molecular and biochemical changes associated with cancer stem cells is essential for the development of targeted therapies. We have reported that CR colon cancer cells, that are enriched in CSCs/CSLCs, readily form spheroids and exhibit increased expression of miR-21, that has been shown to be upregulated in many malignancies, including colon cancer [12], [25]. Expression of miR-21 in the colonic mucosa has also been found to increase during aging, when the incidence of colorectal cancer rises sharply [26]. Although the precise role of miR-21 in the progression of colon carcinogenesis is not fully understood, we reported miR-21 to induce stemness in colon cancer cells by downregulating TGFβ–receptor [26].

Results of our current investigation demonstrate that the colonospheres, that are highly enriched in CSCs/CSLCs with increased expression of miR-21 [12], [22], exhibit decreased expression of the tumor suppressor protein PTEN, a target of miR-21. Our current data also show an inverse relationship between miR-21 and PTEN and supports the contention that miR-21 regulates the expression PTEN. The loss of PTEN, observed in miR-21 over-expressing colon cancer cells could partly be responsible for activation of Akt, as evidenced by the increased levels of pAkt in colonospheres. Increased activation of Akt signaling pathway could play a pivotal role in acceleration of growth of xenograft of miR-21-overexpressing colon cancer cells in SCID mice, that we reported earlier [12] and could confer drug resistance to different malignancies including colorectal cancer [5], [6], [7], [8].

miR-21 the potential “oncomir” invoked us to search for agent(s) that could be effectively used to downregulate miR-21 in colon cancer cells and thus reducing their tumorigenic potential. Rationale for pursuing this line of investigation comes from our recent observation that downregulation of miR-21 in chemo-resistant colon cancer cells causes differentiation and rendering them susceptible to anti-cancer agents, specifically difluorinated curcumin (CDF), an analog of the dietary ingredient curcumin with greater bioavailability than the parent compound [13], [14], [27]. Recent studies from this laboratory have also demonstrated that CDF inhibits the growth of CR colon cancer cells and also upregulates the expression of miR-34, which is downregulated in colon tumors [20]. In addition, earlier studies have demonstrated that CDF to modulate the expression of miR-21 and PTEN in pancreatic cancer [18]. Hence, CDF could be considered as a potential therapeutic agent for both pancreatic and colorectal cancers. Moreover, our observation that CDF modulates miR-21-PTEN-Akt axis in both p53-positive and p53-negative colon cancer cells suggests that CDF could be therapeutically effective in p53-positive and negative colon tumors. Interestingly CDF is also found to be effective in restoring PTEN levels in the metastatic colon cancer cell line SW620. Taken together, the results suggest that CDF could be an effective therapeutic agent for different stages of colon cancer. Addtionally, the fact that CDF modulation of miR-21-PTEN-Akt axis could be observed in chemo-resistant colon cancer cells that are highly enriched in CSCs/CSLCs suggests that this novel analog of curcumin could also be therapeutically effective for recurrent cancer, which is known to be resistant to conventional chemotherapy [14].

In conclusion our current data demonstrate that miR-21 and PTEN are inversely related in that an increased expression of miR-21 results in reduction in PTEN that leads to activation of Akt signaling. These events may play pivotal roles in tumor progression. Furthermore, our observation that miR-21 mediated PTEN-Akt signaling could be normalized by CDF in chemo-resistant colon cancer cells suggests that CDF could be a potential therapeutic agent for recurrent colon cancer.
